# Systemic and Ocular Anti-Inflammatory Mechanisms of Green Tea Extract on Endotoxin-Induced Ocular Inflammation

**DOI:** 10.3389/fendo.2022.899271

**Published:** 2022-07-15

**Authors:** Kai On Chu, Kwok Ping Chan, Yolanda Wong Ying Yip, Wai Kit Chu, Chi Chiu Wang, Chi Pui Pang

**Affiliations:** ^1^ Department of Ophthalmology and Visual Sciences, The Chinese University of Hong Kong, Hong Kong, Hong Kong SAR, China; ^2^ Department of Obstetrics and Gynaecology, the Chinese University of Hong Kong, Hong Kong, Hong Kong SAR, China; ^3^ Li Ka Shing Institute of Health Science, the Chinese University of Hong Kong, Hong Kong, Hong Kong SAR, China

**Keywords:** green tea extract, ocular inflammation, metabolomics, endotoxin induced uveitis, LCMS (liquid chromatography-mass spectrometry)

## Abstract

**Introduction:**

Green tea extract (GTE) alleviated ocular inflammations in endotoxin-induced uveitis (EIU) rat model induced by lipopolysaccharide (LPS) but the underlying mechanism is unclear.

**Objectives:**

To investigate the systematic and local mechanisms of the alleviation by untargeted metabolomics using liquid chromatography-tandem mass spectrometry

**Methods:**

Sprague-Dawley rats were divided into control group, LPS treatment group, and LPS treatment group treated with GTE two hours after LPS injection. The eyes were monitored by slip lamp and electroretinography examination after 24 hours. The plasma and retina were collected for metabolomics analysis

**Results:**

In LPS treated rats, the iris showed hyperemia. Plasma prostaglandins, arachidonic acids, corticosteroid metabolites, and bile acid metabolites increased. In the retina, histamine antagonists, corticosteroids, membrane phospholipids, free antioxidants, and sugars also increased but fatty acid metabolites, N-acetylglucosamine-6-sulphate, pyrocatechol, and adipic acid decreased. After GTE treatment, the a- and b- waves of electroretinography increased by 13%. Plasma phosphorylcholine lipids increased but plasma prostaglandin E1, cholanic metabolites, and glutarylglycine decreased. In the retina, tetranor-PGAM, pantothenic derivatives, 2-ethylacylcarinitine, and kynuramine levels decreased but anti-oxidative seleno-peptide level increased. Only phospholipids, fatty acids, and arachidonic acid metabolites in plasma and in the retina had significant correlation (p < 0.05, r > 0.4 or r < -0.4).

**Conclusions:**

The results showed GTE indirectly induced systemic phosphorylcholine lipids to suppress inflammatory responses, hepatic damage, and respiratory mitochondrial stress in EIU rats induced by LPS. Phospholipids may be a therapeutic target of GTE for anterior chamber inflammation

**Graphical Abstract:**
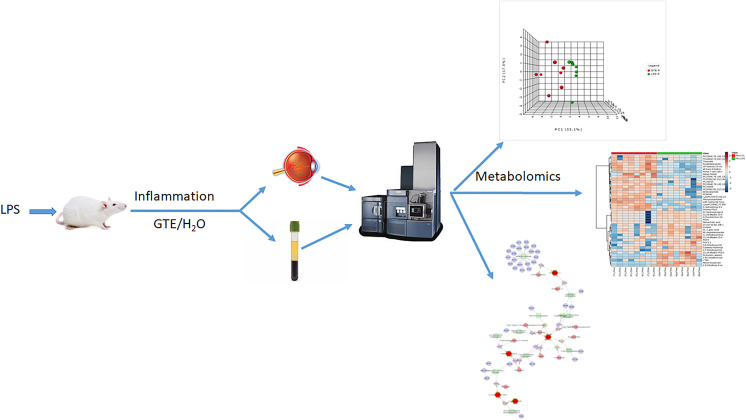


## Introduction

Green tea extract (GTE) possesses potent anti-inflammatory and anti-oxidative properties attributed to its biologically active polyphenol constituents, mainly catechins (1). We have shown its anti-inflammatory effects in ocular tissues of rats with lipopolysaccharide (LPS) induced uveitis model (EIU) (2). Uveitis is a complex inflammatory disease that can affect many parts of the eye and lead to severe irritations, visual dysfunction, and even blindness (3, 4). Uveitis can be caused by infectious agents and autoimmunity, but most uveitis patients are idiopathic. Current therapies mainly rely on antibiotics, corticosteroids, antimetabolites, or cytotoxic agents (5). The latter are immunosuppressive and not suitable for many patients. Corticosteroids are effective for acute and chronic treatments of non-infectious ocular inflammation but with possible side effects, including cataract formation, intraocular pressure elevation (6, 7), hypertension, Cushing’s syndrome, and osteoporosis (8), especially after long term treatment for chronic and recurrent ocular inflammation. In addition, some uveitis patients do not respond to corticosteroids. GTE catechins have been shown to alleviate ocular inflammations in EIU rats (2, 9) and experimental autoimmune uveoretinitis (EAU) mice (10). Its therapeutic potential is to be explored.

The EIU rat model shows intraocular inflammatory responses at anterior uvea and posterior segments that mimics acute infectious uveitis in human (11–13). Characteristic features of EIU rats include cellular infiltration and intense blood vessel dilation in the iris and the retina. The inflammatory responses had been reported to occur at the 4^th^ hour after LPS injection and reach the peak at the 24^th^ hour (13, 14). Pro-inflammatory mediators, like nitric oxide (NO) and prostaglandin (PGE_2_) (15, 16), and intercellular adhesion molecule (ICAM-1) (17), had been found in the inflamed eyes of EIU rats. Pro-inflammatory cytokines tumor necrosis factor-α (TNF–α), interleukin 6 (IL-6), and monocyte chemoattractant protein-1 (MCP-1) were elevated in the anterior chamber. Phosphorylation of nuclear factor-κB (NF-κB) and activation of cluster of differentiation 14 (CD-14) and toll-like receptor 4 (TLR-4) receptors had been detected in the iris and ciliary body (2). In the retina, there were reported increases of TNFα, IL-1β, IL-6, and MMP-9, phosphorylation of NF-κB and signal transducer and activator of transcription 3 (STAT3) in association with 67 kDa laminin receptor (67LR) (9). GTE catechins suppressed the expressions and phosphorylation of these inflammatory proteins, subsequently mediated related metabolic pathways in the cells (18). In this study, we used this model to investigate the underlying mechanisms of how GTE relieve LPS induced inflammation using untargeted metabolomics.

In human endothelial cells with vascular-endothelial-growth factor, after GTE treatment, we found that GTE suppressed cell growth through a wide range of metabolic pathways (19). There was activation of signaling ceramide lipids, antioxidants like ascorbic acid and cysteine, and the energy production vitamin B6 pathway. GTE suppressed nucleotides, the signaling molecule myoinositol, oxidative phosphorylated metabolite succinic acid, and energy production metabolites vitamin B3 and B5. GTE also contributed to homeostatic balance with minimal detrimental pro-oxidative and apoptotic effects. It reportedly protected mouse liver tissues against hepatotoxicity by normalizing blood levels of the energy associated molecules oleoylcarnitine and palmitoylcarnitine, glucose, glutathione, cholic acid, and taurine (19). However, the pharmacodynamic mechanisms of GTE on systemic metabolism and subsequent local anti-inflammation effects on ocular tissues have not been fully elucidated. In this study, we used the metabolomic profiling strategy to investigate the pathological responses involved in the systemic and ocular inflammation induced by LPS in EIU rats and their subsequent suppressions by GTE. We hypothesized that GTE indirectly alleviated ocular inflammation through systemic rectification rather than directly acting on the ocular tissues.

## Materials and Methods

### Materials

Green tea extract (GTE), Theaphenon E^®^, kindly provided by Dr. Yukihiko Hara, contained 70% epigallocatechin gallate (EGCG), 5% epigallate catechins (EGC), 4% epicatechin (EC), 0.6% gallocatechin (GC) and had been assessed in our laboratory (20). The optimal dose, 550 mg/kg, for anti-inflammatory effects against the EIU has been verified (21).

### Endotoxin-Induced Uveitis and GTE Treatment

All experiments were conducted according to the Association for Research in Vision and Ophthalmology (ARVO) statement on the use of animals. Ethics approval for this study was obtained from the Animal Ethics Committee of the Chinese University of Hong Kong. Female Sprague-Dawley rats (about 250 g, 6–8 weeks old) were obtained from the Laboratory Animal Service Centre of Chinese University of Hong Kong. All animals were housed at 25°C with 12/12 hour light-dark cycles and were allowed to access food and water freely. After overnight fasting and body weights were recorded, EIU was induced by injection of lipopolysaccharide (LPS; *Salmonella typhimurium;* Sigma Chemicals, St. Louis, MO) in sterile saline at 1 mg/kg into a footpad of the rats as previous study (2). In brief, the rats were randomly divided into three groups (n=6): i) control group - footpad injected with saline and oral feeding with water; ii) LPS group - footpad injected with 1 mg/kg LPS and oral feeding with water; iii) GTE group – footpad injected with 1 mg/kg LPS and oral feeding with 550 mg/kg GTE 2 hours later. Twenty-four hours after LPS injection, rats were anesthetized and terminated by taking blood through heart puncture and cervical dislocation. Plasma and retina samples were collected for metabolomics analysis.

### Ophthalmic Examinations

Both eyes of the rats were evaluated using a slit lamp as described previously (2). Inflammatory responses including dilation of iris, conjunctival vessels, and iridal hyperemia in the anterior chamber were recorded.

### Electroretinography

Three rats in each group were dark-adapted at least 12 hours before electroretinography (ERG). The rats were anesthetized, and pupils were dilated for ERG examination. The amplitude of the b-wave was measured from the trough of the a-wave to the peak of the b-wave in both eyes from each treatment group.

### Assays for Phospholipase A2 (PLA2) Activity in Plasma

PLA2 activities in the plasma of rats were assayed by a PLA2 kit (Abcam, Cambridge, UK) according to the manufacturer’s instructions.

### Plasma and Retina Samples for Metabolomics Studies

The extraction protocols were modified from reported XCMS protocols (22). In brief, blood was withdrawn from heart puncture and centrifuged at 3,000 g for 20 minutes at 4°C. The upper plasma layer was collected and stored at -80°C. For analysis, 100 µL plasma was mixed with 800 µL ice-cold acetonitrile/methanol (1:1), vortexed for 30 seconds, sonicated for 10 minutes in ice, and centrifuged for 15 minutes at 17,000 g at 4°C. The supernatant was vacuum evaporated, the resultant residue dissolved in 50 µL 50% acetonitrile, vortexed, and centrifuged. The reconstituted supernatant was ready for nano-UPLC-MS analysis.

Retina from the sacrificed rats was washed, dried, and snap-frozen by liquid nitrogen for storage at - 80°C. Prior to analysis, it was smashed into powder in liquid nitrogen. The sample molecules were extracted by 80% methanol at -20°C with vortex, sonication in ice, and centrifugation. The supernatant was vacuum evaporated, and the residues dissolved in 50 µL 50% acetonitrile and centrifuged. The reconstituted supernatant was taken for total protein assay (Pierce BCA protein assay kit, Rockford, AZ) and UPLC-MS analysis (**Supplementary Protocols**).

### Liquid Chromatography-Mass Spectrometry Analysis

Hydrophobic analysis was conducted on an ACQUITY nano UPLC M-Class System by reverse-phase separation on a 75 µm × 250 mm × 1.7 µm BEH130 C18 column (Waters, Milford, MA) at 45°C. Hydrophilic analysis was performed on Agilent 1100 micro HPLC system (Santa Clara, CA) and capillary column EX-nano Inertsil CN-3 (GL Sciences, Tokyo, Japan) at 35°C. Metabolites were detected by Micromass Q-Tof Micro mass spectrometry (Waters MS Technologies, Manchester, UK) in both positive and negative ionization modes.

For hydrophobic analysis, 1 µL sample (injection volume adjusted according to the total protein content) was injected at 0.5 μL/min. Mobile phase A was 5% (v/v) acetonitrile in 0.2% acetic acid, and mobile phase B was 95% (v/v) acetonitrile in 0.2% acetic acid. Binary elution gradient was over a duration of 35.5 min: 0 - 1.34 min, 95% A; 1.34 - 15.0 min, 80% A; 15.0 – 18.5 min, 45% A; 18.5 – 20.5 min, 20% A; 20.5 – 30.5 min, 20% A; 30.5 – 32.5 min, 95% A; 32.5 – 35.5 min, 95% A. For hydrophilic analysis, 1 μL sample was injected at 1.5 μL/min. Mobile phase A was 5% (v/v) acetonitrile in 0.2% acetic acid and mobile phase B was 95% (v/v) acetonitrile in 0.2% acetic acid. The gradient elution was 40.0 min: 0 - 5.0 min, 100% B; 5.0 – 15.0 min, 70% B; 15.0 – 18.0 min, 40% B; 18.0 – 21.0 min, 30% B; 21.0 – 24.0 min, 5% B; 24.0 – 32.0 min, 5% B; 32.0 – 33.0 min, 100% B; 33.0 – 40.0 min, 100% B. The procedure was validated according to published studies (23–25) (**Supplementary Protocols**).

### Electrospray (ESI) Mass Spectrometry (MS)

Metabolites were analyzed by positive and negative ionization modes with Nano Lock spray. In positive mode the capillary voltage was 2,800 V, sample cone 35 V, extraction cone 3.0 V, source temperature 130°C, and cone gas rate 80 L/hr. Full scan was 50 – 1,000 m/z, scan time 1s and inter-scan delay 0.1s. The microchannel plate (MCP) detector was at 2,500 V. Sodium cesium iodide was used for calibration. In negative ESI mode, capillary voltage was -3,500 V, sample cone 35 V, extraction cone 3.0 V, source temperature 120°C, and cone gas rate 80 L/hr. The full scan was 50 – 1,000 m/z, scan time 1s, and interscan delay 0.1s. MCP detector was 2,900 V. All operating parameters were optimized for sensitivity and resolution. Leucine encephalin was used for lock spray calibration. Target analysis by exact mass analysis with target mass ± 0.005 m/z quantified the ionization species of target metabolites and minimized interferences to the validated analysis. The MS/MS profiles of key metabolites were compared with commercially available standards or public databases mainly from XCMS using the above-mentioned LC method with collision energy profile from 20 – 60 eV. Analysis of samples was repeated thrice. The stability of the MS signal intensity over different batches of samples was assessed by selected QC biomarkers from a mixture of control, LPS, and GTE treatment samples for every 12-samples analysis. Stability of QC samples was assessed by the reproducibility (coefficient of variance, CV) of the ion signals (**Supplementary Protocols**).

### Metabolites Investigation

MS data of each sample was processed and compared among the study groups by Markerlynx™ 4.1 (Waters). Metabolites and their adducts were identified by mass (m/z) +/- 10 ppm through the public database METLIN (http://metlin.scripps.edu/) and HMDB (http://www.hmdb.ca/). Identities of metabolites were confirmed by MS/MS fragmentation characteristics from public databases or commercially available standards.

We conducted pathway analysis by METLIN, KEGG (http://www.kegg.com/), Metaboanalyst 3.0 (http://www.metaboanalyst.ca/), IMPaLA (http://impala.molgen.mpg.de/), and Metscape in Cytoscape 3.4. MS data of each sample was processed and compared among the study groups by Markerlynx™ 4.1 (Waters) with ApexTrack peak integration. For extended analysis, S-Plot – P (loadings) was used. The ion counts of each feature were normalized to the total ion intensity. Multivariate analysis and orthogonal partial least square discriminant analysis (OPLS-DA) were conducted. Principle component analysis between groups was performed by Metaboanalyst. Samples were included for analysis if they were within the 95% Hotelling’s T2 range, VIP value > 2, R2 > 0.6, and Q2 > 0.5. Markers were selected from the S-Plot above 0.4 of the P (correlation), and *p* < 0.05 by student t-test. Analysis was repeated if fold changes were more than 1.5 or less than 0.75. Fold changes (FC) between control and LPS groups were calculated as the ion count from the control group divided by the ion count from the LPS group. Fold changes between LPS and GTE groups were calculated similarly. For very highly expressed metabolites, > 1,000 fold changes was assigned. If the metabolites were not detected, the fold change would be assigned as < 0.01 folds (**Supplementary Protocols**).

### Statistical Analyses

Fold changes of metabolites from each pair of treatments were compared by independent t-test. Supervision heatmap clustering of metabolites between the control group and LPS group, and between the LPS group and the GTE group, were evaluated by Metaboanalyst using Euclidean and Ward as the clustering parameters. Data were processed by the normalized sum of response, log scale transformation, and Pareto scaling before statistical analysis by Metaboanalyst. Principal component analysis (PCA) by Metaboanalyst was used to differentiate the samples under different treatments. Statistical significance was defined as *p* < 0.05. Averaged parameters were presented as means ± SD.

## Results

### Clinical Manifestation

**Figure 1:**
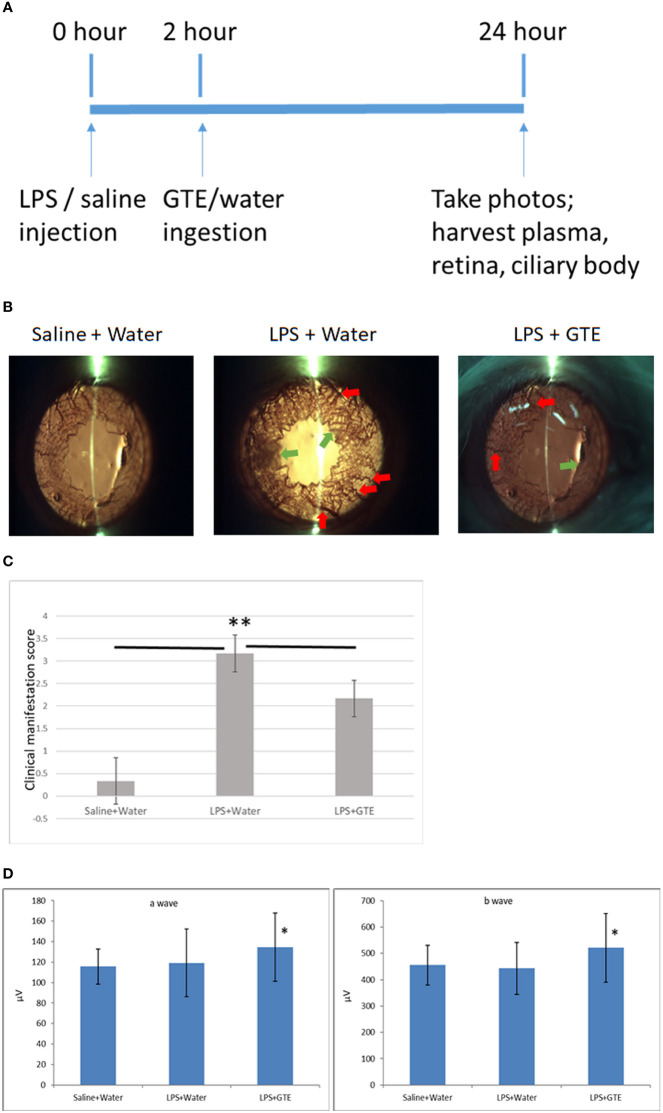
Clinical features of anterior inflammation in the rat eyes. **(A)** Schematic diagram of the experimental procedures. **(B)** Control rats (saline+water ingestion) showed no inflammatory manifestation by slit-lamp examination. Iris of LPS treated EIU rats 24 hours after LPS injection (LPS+water) showed hyperemia (red arrow) and edema (green arrow). EIU rats treated by GTE two hours after LPS injection (LPS+GTE) showed suppression of inflammatory responses. **(C)** Clinical manifestation score of ocular inflammation in normal control rats (saline+water), EIU (LPS+water) rats, and EIU rats treated by GTE (LPS+GTE). n=6 in each group. Error bar indicates standard deviation. **—**: comparison between two groups. ** - p < 0.05 comparing with different treatment groups by Mann–Whitney U test. **(D)** Inter-group comparisons of a-wave and b-wave of scotopic ERG amplitudes. Rats were tested 24 hours after LPS injection. The asterisk marked the statistical significance of the comparison between the three groups. Data are also presented as mean ± SD and analyzed by ANOVA test (*P < 0.05, n = 3; both eyes from each rat).

Similar to our previous study (2), slit-lamp examination showed inflammatory features in the iris, including hyperemia and edema associated with miosis and fibril formation, which appeared 24 hours after LPS injection but were resolved after GTE ingestion at 2 hours thereafter (**Figures 1A–C**). Clinical scores were increased by LPS induction (0.33 ± 0.52, compared to negative control 3.2 ± 0.41, P < 0.05) and reduced after GTE treatment (2.17 ± 0.41) (P < 0.05). The conductivity of rod photoreceptors and bipolar cells of the retina were not affected by LPS as shown by scotopic ERG (a-wave = 119.1 ± 33.1, b-wave = 443.3.3 ± 98.9, as compared to negative control, a-wave = 115.7 ± 17.3; b-wave = 455.3 ± 75.5), which, however, was slightly increased by 13% (P < 0.05) following GTE treatment (a-wave = 134.4 ± 33.4, b-wave = 521.7 ± 130.5) (**Figure 1D**).

### Differentiation of Metabolites in Plasma and Retina After Treatments

**Figure 2:**
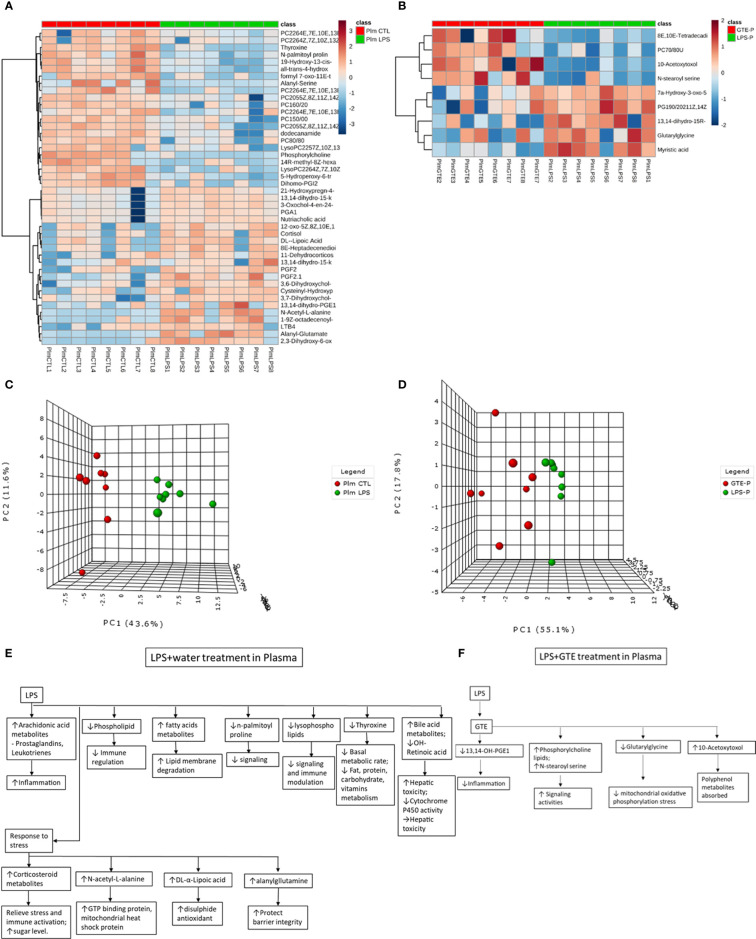
Heatmaps of metabolites showed patterns of differential metabolite profiles between different treatment groups in plasma. **(A)** Between controls and EIU as induced by LPS in the plasma; **(B)** Between EIU induced by LPS and after GTE treatment in the plasma Principal component analysis (PCA) showed **(C)** comparison of controls and EIU induced by LPS, **(D)** comparison of EIU induced by LPS and after GTE treatment samples in plasma. Metabolites and their associated biological activities were shown in **(E)** the plasma of the EIU rats induced by LPS, and **(F)** the plasma of the EIU rats after GTE treatment.

**Figure 3:**
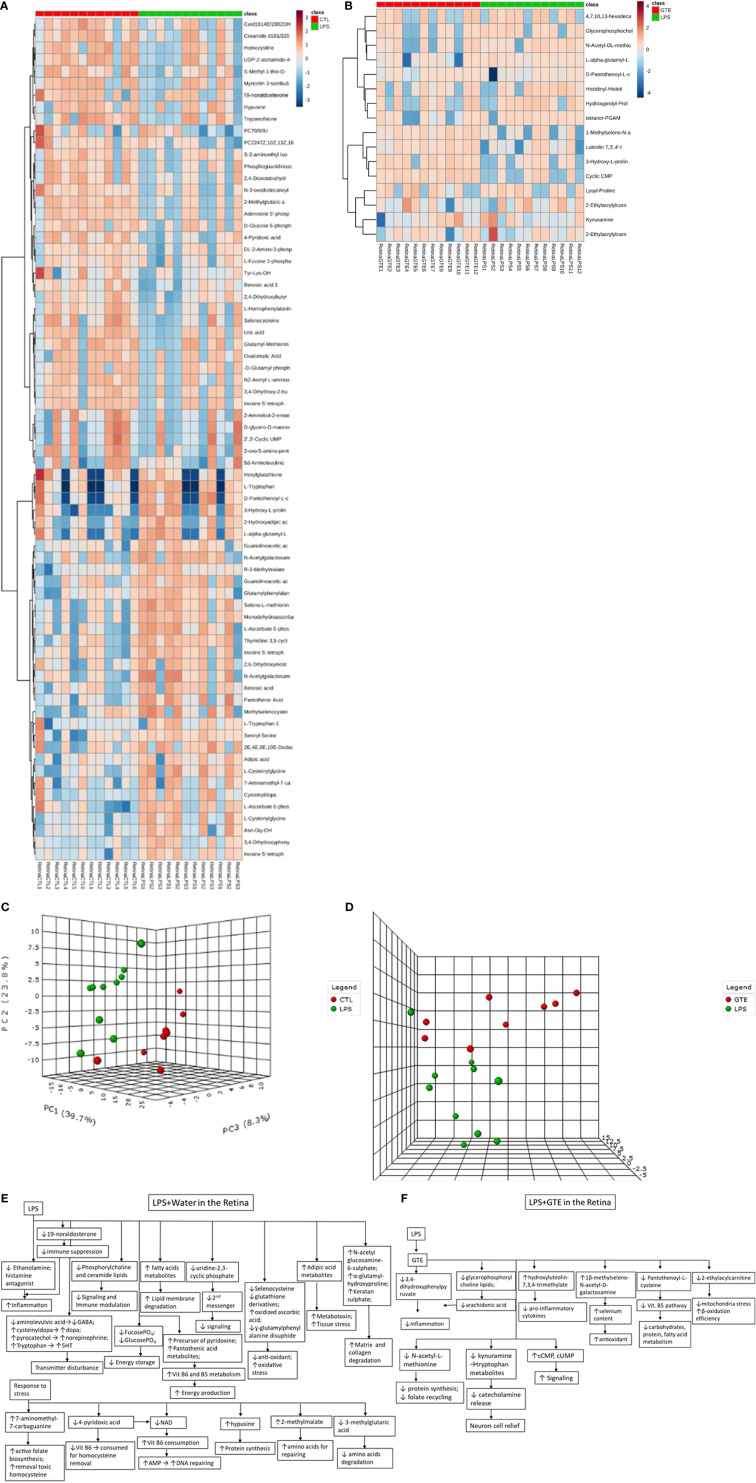
Heatmaps of metabolites showed patterns of differential metabolite profiles between different treatment groups in retina. **(A)** Between controls and EIU as induced by LPS in the retina; **(B)** Between EIU induced by LPS and after GTE treatment in the retina. Principal component analysis (PCA) showed **(C)** comparison of controls and EIU induced by LPS samples in the retina, and **(D)** comparison of EIU induced by LPS induction and after GTE treatment samples in the retina. Metabolites and their associated biological activities were shown in **(E)** the iris ciliary body of the EIU rats after GTE treatment, **(E)** the retina of EIU induced by LPS, and **(F)** the retina of EIU after and GTE treatment.

Differential patterns of metabolites were found in plasma and retina samples among different treatment groups (**Supplementary Tables 1A, B**). LPS treatment led to decrease in plasma lipids, fatty acids and tyrosine metabolites but increase of arachidonic acid, retinoic acid, corticosteroid, cholestanoic acid, peptides, amino acids, and antioxidants. After GTE treatment, metabolites of lipids, fatty acids, and polyphenol increased while metabolites of arachidonic acid, cholestanoic acid, peptides, and amino acids decreased. In the retina, metabolites of phospholipids, histamine, corticosteroids, and nucleotides decreased but those of vitamins, co-factors, acylcarnitines, and neurotransmitters increased. GTE treatment decreased metabolites of lipids and fatty acids, acylcarnitine, peptides, amino acids, vitamins, co-factors, arachidonic acid, and endogenous antioxidant, but increased those of cyclic nucleotides. Heatmaps showed metabolites related to inflammation, oxidation, signaling, energy suppression, and structural degradation were associated with LPS whereas metabolites related to anti-inflammation, anti-oxidation, signaling activation, energy recovery, and structural molecule synthesis were associated with GTE. Principal component analysis (PCA) showed a clear separation of metabolomic profiles of the samples among different treatments (**Figures 2**, **3**).

### Pathways Analysis Following LPS and GTE Treatments in Plasma and Retina

Metscape analysis showed that systemic and retinal pathways affected by LPS induction were associated with inflammation, signaling, anti-oxidation, energy production, and biosynthesis. GTE affected systemic and local metabolic pathways involved in inflammation, signaling, energy production, and biosynthesis (**Supplementary Table 2**).

In plasma, pathways activated by LPS were associated with inflammation, such as steroid hormones, prostanoids, and arachidonic acid. Pathways related to signaling such as glycerophospholipid and tyrosine metabolism were depressed. Metabolites related to mitochondrial acitivity, and lipoate metabolism increased. After GTE administration, the systemic pathways associated with arachidonic acid and linoleate metabolism, and fatty acid biosynthesis were depressed while signaling mechanism of glycerophospholipid was activated.

In the retina, LPS induced inflammation-related prostaglandin formation pathway, amino acids metabolism, and vitamin B5 metabolism. LPS modulated energy-related pathways. It depressed fructose, mannose, and vitamin B6 metabolisms but activated vitamin B5-CoA biosynthesis. It depressed signaling related pathway (glycerophospholipid metabolism), anti-oxidation pathway (selenoamino acid metabolism), and nucleotides biosynthesis pathway (pyrimidine metabolism). The pathways activated in the retina following GTE administration were associated with inflammation-modulating signaling pathway (glycosphingolipid biosynthesis) and nucleotide metabolism pathway (pyrimidine metabolism). Inflammation was relieved as shown in the deactivation of prostaglandin formation from arachidonate metabolism, membrane formation (glycerophospholipid biosynthesis), anti-oxidation pathway (methionine and cysteine metabolism), and energy production pathway (vitamin B5 - CoA biosynthesis and fatty acid beta oxidation).

Metscape also showed interconnections of pathways of inflammatory mediators, such as prostaglandins, corticosteroids, leukotrienes, arachidonic acids (**Supplementary Figure 1**). Immune-related prostanoids and leukotriene metabolites were elevated whereas the 5-hydroperoxide (5-HPETE), a precursor of leukotriene, was reduced (**Supplementary Figure 2**). LPS also depressed the tyrosine pathway associated with thyroxine biosynthesis (**Supplementary Figure 3**).

### Correlation of Metabolites in Plasma With Retina

**Figure 4:**
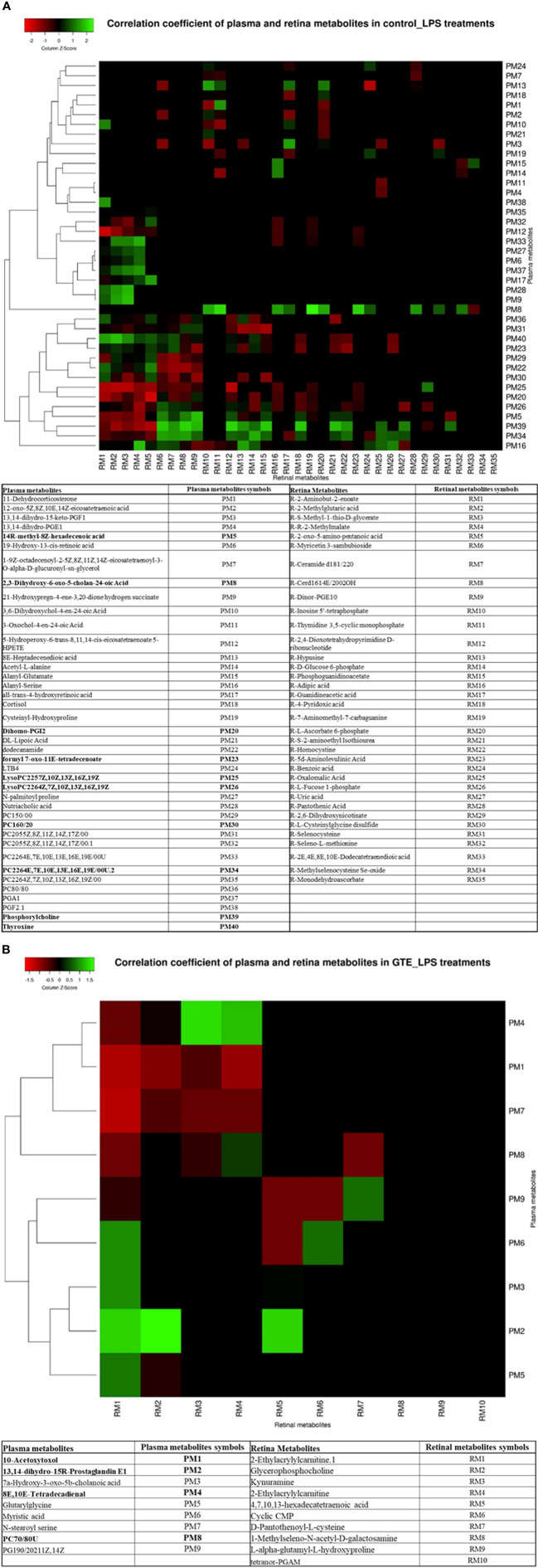
Heatmap showing the correlation coefficients between systemic metabolites in the plasma associated with metabolites in the retina of EIU rats before and after GTE treatment. The labeled metabolites are the plasma metabolites that gave correlations (r > 0.5 or r < -0.5; p < 0.05) with metabolites in the retina. The heatmaps show the correlation coefficients between systemic metabolites regulation associated with **(A)** metabolites in the retina under LPS induction, and **(B)** metabolites in the retina under GTE treatment. The tables show the labeled metabolites corresponding to the metabolites present in the plasma and the retina. The bold plasma metabolites in the tables indicate that those metabolites give higher correlation coefficients and have wider coverage to the metabolites present in the retina.

We correlated the differentially expressed metabolites in the plasma and retina to explore the relationships between metabolites expression in the local ocular tissue and those in circulation. Plasma phospholipids, fatty acid metabolites and bile acid metabolites were highly correlated (p < 0.05; r > 0.4 or r < -0.4) with many metabolites in the retina (**Supplementary Table 3**) (**Figure 4**). After LPS induction, phosphochloline lipid metabolites, PC (22:6 (4E,7E,10E,13E,16E,19E)), lysophosphochloline lipid metabolites, lysoPC (22:6(4Z,7Z,10Z,13Z,16Z,19Z)), and fatty acid metabolite, 14R-methyl-8Z-hexadecenoic acid in the plasma were negatively correlated to 5, 6, 9, and 6 metabolites but were positively correlated to 17, 15, 5, and 13 metabolites in the retina respectively. After GTE treatment, phosphocholine lipid metabolites, PC (7:0/8:0), phospholipid, PG (19:0/20:2(11Z,14Z)) and fatty acid metabolites, N-stearoylserine, in the plasma were negatively correlated to 4, 3, and 4 metabolites but were positively correlated to 0, 1, and 0 metabolite in the retina respectively.

### Comparison of PLA2 Activities

**Figure 5:**
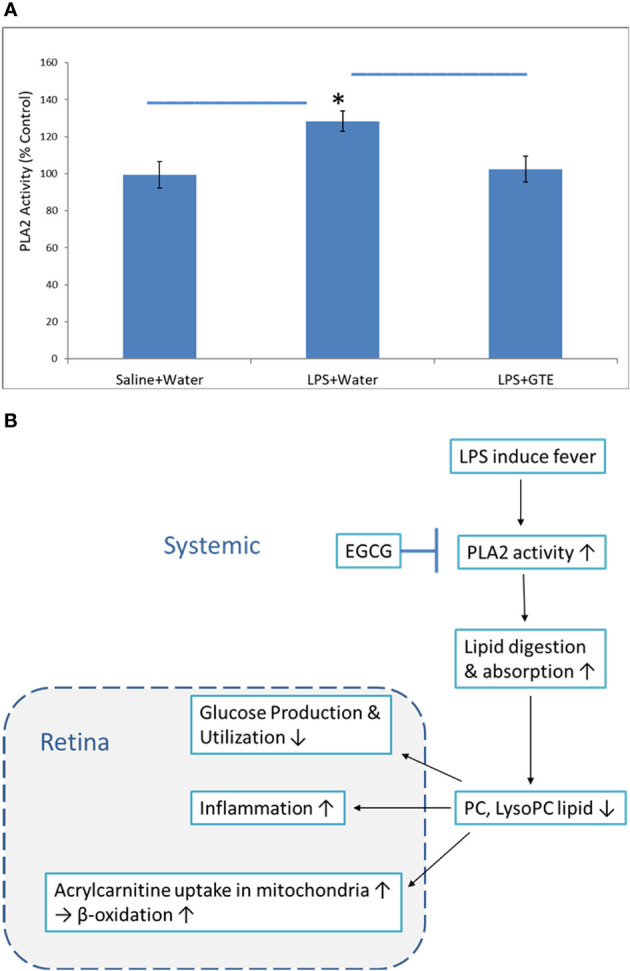
The proposed mechanism of how catechins in GTE inhibit phospholipase A2 (PLA2) activity and alleviate retinal inflammation and metabolic stress. **(A)** The chart shows the relative PLA2 activities in six rat plasma samples of different treatment groups. **(B)** LPS induced fever caused by LPS induced PLA2 activity to increase lipid digestion and subsequently reduce phosphocholine lipids. Catechins inhibited PLA2 activity, increased PC levels, and subsequently inhibited inflammation. *:- a significant difference between groups; error bar:- standard derivation; horizontal bar:- one-way ANOVA comparison between three groups.

Phospholipase A2 (PLA2) hydrolyses the sn-2 position of membrane and plasma glycerophospholipids to release arachidonic acid which is a precursor of eicosanoids such as prostaglandins and leukotrienes for inflammation actions. It also produces lipid mediators like lysophospholipids. The relative PLA2 activities in plasma in the LPS group were 128.3 ± 5.5% higher than the negative control and GTE treatment group, 102.5 ± 5.5% (P < 0.05) (**Figure 5A**).

## Discussion

Inflammation of the iris in our EIU model and the anti-inflammatory effects of GTE treatment were consistent with our previous study (2). In chronic ocular inflammation, neuritis has been reported to detriment neuron conductivity and diminish evoked potential (26). However, in our EIU model of acute ocular inflammation, the conductivity was not affected as shown by ERG in the a- and b-waves. GTE could improve the sensory symptom but not the conductivity of the nerve (27) through anti-oxidation protection (28). In our study, the conductivity of the rod photoreceptors and bipolar cells was improved by GTE treatment (**Figure 1D**), suggesting temporary increase of conductivity by GTE.

Our results showed different systemic and retinal metabolic responses to the LPS induced inflammation and subsequent GTE alleviation. The systemic metabolic responses to LPS included increases in inflammation promoting prostanoids, inflammation resolving corticosteroids, bile acids, endogenous antioxidants, and degraded peptides. There were decreases in structural and signal related lipids and fatty acids, and basal metabolic regulatory hormone thyroid hormone tyrosine. The inflammation was so intensive that 5-hydroperoxide (5-HPETE), a precursor of leukotriene, was exhausted (**Supplementary Figure 2**). The systemic metabolic responses of GTE on LPS induced inflammation included increases in structural and signal related lipids and fatty acids but decreases in inflammation associated prostanoids, bile acids, and degraded peptides. Similarly, the retinal metabolic responses to LPS included increase of energy associated vitamins, co-factors, and metabolites of neurotransmitters but decreases of anti-inflammation related corticosteroids and histamine metabolites, signaling related phospholipids and nucleotide metabolites. The retinal metabolic responses of GTE on LPS induced inflammation included increases in signaling nucleotides metabolites but decreases in structural and signaling related lipid and fatty acid mediators, inflammation associated prostanoids, and energy-related vitamins and co-factors (**Figures 2**, **3**). Taken together, the results suggested that GTE relieved different metabolic and energetic stresses, and resolved structural destruction and toxicity.

Pathway analysis also showed that LPS activated systemic metabolic inflammatory-related pathways, prostaglandin biosynthesis, leukotriene metabolism, and steroid hormone biosynthesis. LPS caused homeostatic responses to release corticosteroids to counter inflammation. In the retina, it activated inflammation pathways, caused protein degradation, and disturbed energy production metabolisms by increased vitamin B5 but decreased vitamin B6, fructose and mannose metabolisms pathways. It also suppressed pathways involving selenoamino acid metabolism to reduce antioxidant capacity and caused oxidative stress. GTE relieved systemic inflammation by suppressing systemic inflammation-related pathways that involved arachidonic acid metabolism and activating cellular signaling glycerophospholipid metabolism. In the retina, it relieved inflammation by activating signaling pathways like the glycosphingolipid biosynthesis pathway and suppressing prostanoid production and glycerophospholipid biosynthesis for membrane signaling (**Supplementary Table 2**).

Significant differentiated metabolite profiles in the plasma were found following LPS induction and after GTE treatment. Phosphorylcholine lipids have anti-inflammatory property and inhibited TNF-α-mediated NF-κB translocation in a dose-dependent manner in Caco-2 cells (29). Classical eicosanoids from COX mediated metabolites of arachidonic acid and N-acylamides, N-palmitoyl proline, are strongly pro-inflammatory (30). LPS modulates phosphorylcholine lipid, arachidonic acid metabolites, N-palmitoyl proline, thyroxine, and nutriaholic acid, which are associated with inflammation responses (31, 32), basal metabolism, hepatic detriment (33, 34), and signaling (35). Alanylglutamine can reduce infection-induced inflammatory damage, and infection-associated symptoms like dehydration, and inhibits apoptosis due to cellular damage (36). LPS induced homeostatic responses to increase corticosteroids, alanylglutamine, and DL-α-lipoic acid are associated with immune responses (37, 38), mitochondria stress suppression (33), and anti-oxidation recovery (39) (**Figure 2E**) (**Supplementary Table 1A**).

After GTE treatment, it increased phosphorylcholine lipids but lowered free structural lipids, arachidonic acid metabolites, bile acid metabolites, 7alpha-hydroxy-3-oxo-5b-cholanoic acid, and acyl glycine, glutarylglycine, which is associated with mitochondrial fatty acid beta-oxidation as acylglycines, are normally minor metabolites of fatty acids, indicating inflammation was relieved, cellular membrane integrity maintained, hepatic recovered and mitochondria stress relieved (**Figure 2F**) (40). Only polyphenol catabolite, 10-acetoxytoxol, was detected in plasma suggesting extensive metabolism of catechins whose direct anti-inflammatory effects are thus questionable (**Supplementary Table 1B**).

In the retina, LPS lowered the histamine antagonist, ethanolamine and the endogenous antioxidant se-methylselenocysteine but increased the glucocorticoid 19-noraldosterone, the catabolite monodehydroascorbate (41, 42) and tissue damaged associated metabolites, N-acetylglucosamine 6-sulfate and L-alpha-glutamyl-l-hydroxyproline. Homeostatic response increased the absorption of L-ascorbate 6-phosphate, elevated 7-aminomethyl-7-carbaguanine and D-pantothenoyl-L-cysteine (43, 44). But it depleted inflammation mediators, fucose-1-phosphate, and energy sources, D-Glucose-6-phosphate (45), increased membrane signaling lipid metabolites, metabotoxin, adipic acid (46), and increased amino acid precursor, R-2-methylmalate. Neuron activity was compromised as the reduction of neural peptide, N-acetylaspartylglutamic acid, amino acid, aminolevulinic acid, and increasing the melanocytes and neurotransmitters metabolites, cysteinyldopa and pyrocatechol (47, 48). These findings indicate inflammation; oxidative, mitochondrial and retinal stress; and tissue damage have occurred with the activation of energy production and biosynthesis pathways (**Supplementary Tables 1C, D**) (**Figure 3E**).

After GTE treatment, the metabolites profiles in the retina also showed inflammation resolved. GTE reduced the inflammatory prostaglandin metabolite tetranor-PGAM and lowered free glycerophosphocholine level (49). It also decreased the collagen degradant L-alpha-glutamyl-l-hydroxyproline, elevated the selenium antioxidant 1β-methylseleno-N-acetyl-D-galactosamine, decreased antioxidant amino acid metabolites, N-acetyl-L-methionine and energy production associated metabolites, D-pantothenoyl-L-cysteine, and lowered 2-ethylacrylcarnitine and kynuramine. But it increased signaling cyclic CMP (50). L-Carnitine transports fatty acids into the mitochondrial matrix to produce energy. L-carnitine and its esters metabolites increased during oxidative stress (51). Kynuramine is a biogenic amine, which is a major metabolite of melatonin in the brain produced by oxidative and photochemical reactions. Therefore, they can be a biomarker for oxidative stress and inflammation (52). It indicates relief of inflammation and oxidative, mitochondrial, and neuronal stress with recoveries of membrane integrity, cellular activities, and biosynthesis. Presence at a low level of the polyphenol metabolite, 6-hydroxyluteolin-7,3’,4’-trimethyl ether, in the retina indicated low bioavailability and extensive metabolism of catechins (**Figure 3F**) (53).

Correlation of the metabolites in the plasma with the retina showed the highest correlated systemic metabolites to retinal metabolites were phosphorylcholine lipids, fatty acids, and arachidonic acid metabolites (**Figure 4**) (**Supplementary Table 3**). The phosphorylcholine lipids involve immune regulation and maintenance of tissue homeostasis. Bioactive endogenous lipids are important mediators in all phases of inflammation involving regulation, fine-tuning, and cessation. While classical eicosanoids are mainly pro-inflammation, some lysoglycerophospholipids and sphingolipids are pro-resolving mediators. Phosphatidylcholine (PC) also showed anti-inflammatory properties against TNF-α induced inflammation in ulcerative colitis (29, 31, 32). LPS caused both ocular and systemic inflammation possibly by suppressing phosphorylcholine lipids production. The systemic levels of phosphatidylcholine and lysophosphatidylcholine were correlated with retinal metabolites. On the other hand, GTE increased systemic phosphatidylcholine and sphingosine metabolite, N-stearoyl serine, were negatively associated with retinal metabolites relating to the malfunction of mitochondria, neuron stress, tissue damage, and inflammation (**Figure 4B**) (54, 55). LPS activated toll-like receptor (TLR-4) expression and induced innate immune response leading to uveitis (56). Phospholipids decreased activation of TLR-4 by competitive interaction with accessory proteins of TLR-4 (57). PLA2 increased lipid digestion and reduced absorption of phosphatidylcholine and lysophosphatidylcholine (58, 59). Catechins inhibited PLA2 and could increase phosphatidylcholine and lysophosphatidylcholine levels and decrease the arachidonic acid (AA), a precursor of eicosanoid metabolites, to release for anti-inflammation (60). In this study, LPS lowered the systemic phosphocholine lipid level and indirectly induced inflammation in the retina. GTE could then inhibit TLR-4 activity indirectly by increasing the systemic phospholipid and sphingosine levels (**Figure 5**).

In our metabolomic study, GTE alone was not used as a control because it affects many metabolic pathways (61). This study aimed to investigate the underlying metabolic mechanisms how GTE can resolve the inflammation induced by LPS, so GTE with LPS served as treatment controls while LPS as a positive control. However, this study is an untargeted metabolomics study. Although we have verified the identity of the metabolites according to the exact mass and fragmentation patterns, we need to do targeted metabolomics to further confirm the identity of each metabolite. Since many metabolites are not commercially available, we cannot perform a comprehensive targeted metabolomics analysis in this study. Nevertheless, we plan to select a few target phospholipids for further studies and validation.

## Conclusion

The untargeted metabolomics approach can overview a series of underlying mechanisms of LPS caused inflammation and GTE alleviation simultaneously. It showed LPS induced inflammation involved signaling suppression, oxidative and respiratory stress, and increased energy consumption in the retina. GTE relieved the inflammation was associated with alleviating oxidative, mitochondrial, and neuronal stresses, and energy consumption. GTE may increase systemic phospholipids to alleviate ocular inflammation indirectly rather than act directly with anti-inflammatory effects on the retina tissues. Phospholipids could be a potential therapeutic agent for ocular inflammation.

## Data Availability Statement

The original contributions presented in the study are included in the article/**Supplementary Material**. Further inquiries can be directed to the corresponding author.

## Ethics Statement

The animal study was reviewed and approved by the Animal Ethics Committee of the Chinese University of Hong Kong. Written informed consent was obtained from the owners for the participation of their animals in this study.

## Author Contributions

KOC: conceptualization, methodology, validation, formal analysis, investigation, data curation, writing - original draft, writing - review and editing, visualization, supervision, and project administration; KPC and YY: methodology and investigation; WC: writing - review and editing; CW: review and editing, funding acquisition; CP: writing – review and editing, supervision, resources, and funding acquisition. All authors contributed to the article and approved the submitted version.

## Funding

The study was funded by Research Grant Council General Research Fund (Project No. 475012 to CCW) and Health and Medical Research Fund (Project No. 12130811 to CPP), Hong Kong.

## Conflict of Interest

The authors declare that the research was conducted in the absence of any commercial or financial relationships that could be construed as a potential conflict of interest.

## Publisher’s Note

All claims expressed in this article are solely those of the authors and do not necessarily represent those of their affiliated organizations, or those of the publisher, the editors and the reviewers. Any product that may be evaluated in this article, or claim that may be made by its manufacturer, is not guaranteed or endorsed by the publisher.
