# Supplementary material for: Systemic and Ocular Anti-Inflammatory Mechanisms of Green Tea Extract on Endotoxin-Induced Ocular Inflammation
Source: Front Endocrinol (Lausanne). 2022 Jul 15;13:899271. doi: 10.3389/fendo.2022.899271 (PMC9335207; doi:10.3389/fendo.2022.899271)
Supplement: Supplementary file 1 [file DataSheet_1.docx]

**Supplementary Protocols**

**Plasma and retina samples for metabolomics studies**

Blood was withdrawn from the heart puncture and transferred into a heparin tube. The tubes were vortexed immediately after blood collection followed by centrifugation at 3,000 g for 20 minutes at 4 °C. The upper plasma layer was transferred to a glass tube, snap-frozen by liquid nitrogen, and stored at -80 °C. For analysis, 100 µL plasma was mixed with 800 µL ice-cold acetonitrile/methanol (1:1) mixture in a glass vial, vortexed for 30 seconds, sonicated for 10 minutes in an ice bath, and centrifuged for 15 minutes at 17,000 g at 4 °C. The supernatant was transferred into another glass vial and dried by vacuum evaporation. The residue was dissolved in 50 µL 50% acetonitrile, vortexed, and centrifuged for 15 minutes at 17,000 g at 4 °C. The supernatant was transferred into a glass vial and stored at - 80 °C until nano-UPLC-MS analysis.

Retina samples were obtained from the sacrificed rats and washed with phosphate buffer, dried, snap-frozen by liquid nitrogen, and stored at - 80°C. For analysis, the frozen tissues were smashed into powder with a stainless steel clamp that had been cooled by liquid nitrogen. The powder was immediately mixed with 0.4 mL 80% methanol at -20 °C, vortexed, sonicated in an ice bath, and centrifuged at 17,000 g for 10 minutes. The supernatant was transferred into another glass tube and dried under vacuum evaporation. The protein residues were dissolved in 50 µL 50% acetonitrile and centrifuged for 15 minutes at 17,000 g at 4 °C. An aliquot of the supernatant was used for total protein assay (Pierce BCA protein assay kit, Rockford, AZ). The remaining supernatant was used for UPLC-MS analysis.

**Liquid chromatography-mass spectrometry analysis**

For hydrophobic analysis, a nano-ultra-performance liquid chromatography system (ACQUITY Nano UPLC M-Class System, Waters, Milford, MA) was used for reverse-phase separation by a 75 µm × 250 mm × 1.7 µm BEH130 C18 column (Waters) at 45 °C. About 1 µL sample (injection volume was adjusted according to the total protein content) was injected at a flow rate of 0.5 μL/min. Gradient elution was performed with mobile phase A (5% (v/v) acetonitrile in 0.2% acetic acid) and mobile phase B (95% (v/v) acetonitrile in 0.2% acetic acid) over a duration of 35.5 min on a binary elution gradient: 0 - 1.34 min, 95% A; 1.34 - 15.0 min, 80% A; 15.0 – 18.5 min, 45% A; 18.5 – 20.5 min, 20% A; 20.5 – 30.5 min, 20% A; 30.5 – 32.5 min, 95% A; 32.5 – 35.5 min, 95% A.

Sample molecules were separated on a hydrophilic capillary EX-nano Inertsil CN-3 0.2 mm × 150 mm × 3 μm column (GL Sciences, Tokyo, Japan) by micro high performance liquid chromatography (Agilent 1100 micro HPLC system, Santa Clara, CA) at 35 °C. About 1 μL sample (injection volume was adjusted according to the total protein content of the respective tissue extract) was injected at a flow rate of 1.5 μL/min. Gradient elution was performed with mobile phase A (5% (v/v) acetonitrile in 0.2% acetic acid) and mobile phase B (95% (v/v) acetonitrile in 0.2% acetic acid). The elution duration was 40.0 min on a binary elution gradient: 0 - 5.0 min, 100% B; 5.0 – 15.0 min, 70% B; 15.0 – 18.0 min, 40% B; 18.0 – 21.0 min, 30% B; 21.0 – 24.0 min, 5% B; 24.0 – 32.0 min, 5% B; 32.0 – 33.0 min, 100% B; 33.0 – 40.0 min, 100% B.

**Electrospray (ESI) mass spectrometry (MS)**

Metabolites were analyzed by Q-Tof mass spectrometry (Micromass Q-Tof micro, Waters MS Technologies, Manchester, UK) in both positive and negative ionization modes. The system was controlled and data acquired by the MassLynx 4.1 software (Waters). Nano Lock spray system ESI system was used for the mass spectrometry. The setting was at positive mode: 2,800 V capillary voltage, 35 V sample cone voltage and 3.0 V extraction cone voltage at 130 °C source temperature and 80 L/hr cone gas rate. Full scan was performed at 50 – 1,000 m/z, with scan time 1 s and inter-scan delay of 0.1 s. The microchannel plate (MCP) detector was set at 2,500 V. Sodium cesium iodide was used for calibration. The negative ESI mode conditions: -3,500 V capillary voltage, 35 V sample cone and 3.0 V extraction cone at 120 °C source temperature and 80 L/hr cone gas rate. The full scan was 50 – 1,000 m/z, with 1 s scan time and 0.1 s interscan delay. MCP detector was set at 2,900 V. All other operating parameters were optimized for the best sensitivity and resolution. Leucine encephalin was used for lock spray calibration every 5 minutes and detected at 556.6606 m/z for positive ESI and 554.6147 m/z for negative ESI. Target analysis by exact mass analysis method with target mass ± 0.005 m/z quantified the ionization species of target metabolites and minimized interferences to the validated analysis. The MS/MS profile of key metabolites was compared with commercially available standards or public databases mainly from XCMS using the above-mentioned LC method with collision energy profile from 20 – 60 eV. The analysis sequence of control, EIU, and GTE samples was randomly assigned to each batch of samples. Samples were repeatedly injected in three batches. The stability of the MS signal intensity over different batches of samples was assessed by selected QC biomarkers, which were mixtures of control, LPS, and GTE treatment samples. They were analyzed every 12 samples. The stability of QC samples was assessed by the reproducibility (coefficient of variance, CV) of the ion signals.

**Metabolites investigation**

MS data of each sample was processed and compared among the study groups by Markerlynx^TM^ 4.1 (Waters), which used ApexTrack peak integration for chromatographic peak alignments and detection. The processing parameters were: Analysis type – Peak detection; Initial retention time – 0; Final retention time – 50; Low Mass – 50; High Mass – 1,000; XIC window – 0.01; Apex Track Peak Parameters - No; Noise elimination level – 4; Deisotope data – Yes. For extended analysis, parameters are scale type for x-variable – Pareto; Transformations – automatic; S-Plot – P (loadings); Label – sample group. The ion counts of each feature in the sample were normalized to the total ion intensity for statistical analysis to minimize variations due to the sample injection and ionization procedure. Multivariate analysis and orthogonal partial least square discriminant analysis (OPLS-DA) were conducted. Principle component analysis between groups was performed by Metaboanalyst. Samples were included for analysis if they were within the 95% Hotelling’s T2 range, VIP value > 2, R2 > 0.6, and Q2 > 0.5. Markers were selected from S-Plot above 0.4 of the P (correlation), and *p* < 0.05 by student t-test. Analysis was repeated if fold changes were more than 1.5 or less than 0.75.

Metabolites were identified by the accurate mass method. Metabolite mass (m/z) was searched through the public database METLIN (<http://metlin.scripps.edu/>) and HMDB (http://www.hmdb.ca/) with mass accuracy +/- 10 ppm with different adducts. Identities of metabolites were confirmed by MS/MS fragmentation characteristics from public databases or commercially available standards. Fold changes (FC) between control and LPS groups were calculated as the ion count from the control group divided by the ion count from the LPS group. Fold changes between LPS and GTE groups were calculated as the ion count from the LPS group divided by the ion count from the GTE group. For metabolites highly expressed in one treatment group, the fold changes could be very large and assigned as > 1,000 folds. If the metabolites were not detected, the fold changes would be very small and assigned as < 0.01 folds.

We conducted pathway analysis by METLIN, KEGG (http://www.kegg.com/), Metaboanalyst 3.0 (http://www.metaboanalyst.ca/), IMPaLA (http://impala.molgen.mpg.de/), and Metscape in Cytoscape 3.4.
