# Supplementary material for: Systemic and Ocular Anti-Inflammatory Mechanisms of Green Tea Extract on Endotoxin-Induced Ocular Inflammation
Source: Front Endocrinol (Lausanne). 2022 Jul 15;13:899271. doi: 10.3389/fendo.2022.899271 (PMC9335207; doi:10.3389/fendo.2022.899271)

**Supplementary Figure 3.** Thyroxine formation from tyrosine pathway in the plasma of the EIU rats induced by LPS. Big red hexagon indicates the identified metabolites level increased, whereas small red hexagon indicates the reduced level of the identified metabolites. – enzyme involved in the reaction; - reaction; - gene involved in the reaction.


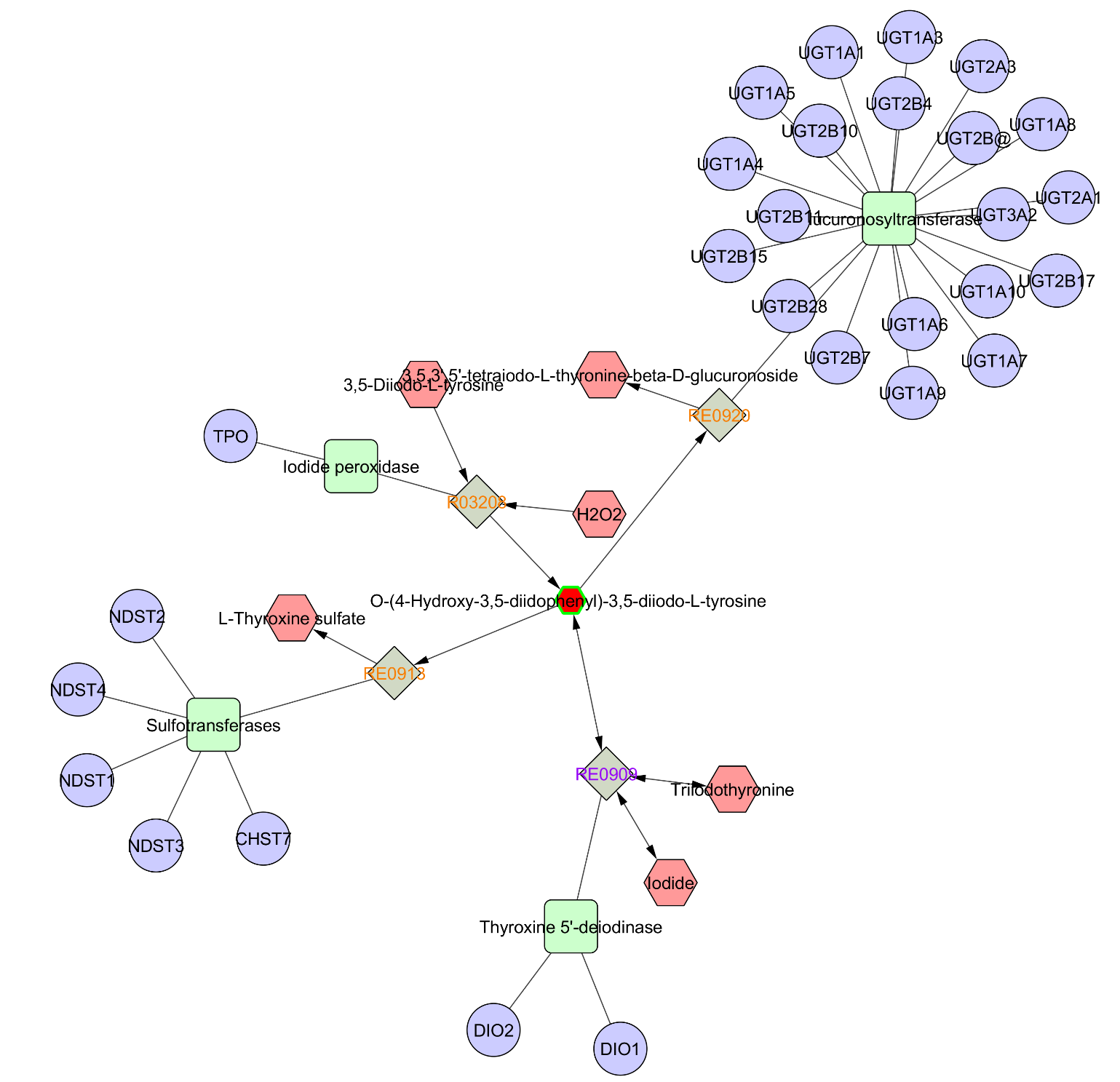

Supplement: Supplementary file 2 [file DataSheet_2.zip › Supplementary Figure 3.DOCX]
