# Supplementary material for: Systemic and Ocular Anti-Inflammatory Mechanisms of Green Tea Extract on Endotoxin-Induced Ocular Inflammation
Source: Front Endocrinol (Lausanne). 2022 Jul 15;13:899271. doi: 10.3389/fendo.2022.899271 (PMC9335207; doi:10.3389/fendo.2022.899271)
Supplement: Supplementary file 3 [file Table_1.docx]

**Supplementary Table 1.** Differential metabolite expressions obtained by orthogonal partial least square discriminant analysis (OPLS-DA): (a) comparing control rats and EIU rats induced by LPS in plasma; (b) EIU rats treated by GTE in plasma; (c) comparing control rats and EIU rats induced by LPS in the retina; and (d) EIU rats treated by GTE in the retina.

(a)

(b)

(c)

(d)
