# Supplementary material for: Systemic and Ocular Anti-Inflammatory Mechanisms of Green Tea Extract on Endotoxin-Induced Ocular Inflammation
Source: Front Endocrinol (Lausanne). 2022 Jul 15;13:899271. doi: 10.3389/fendo.2022.899271 (PMC9335207; doi:10.3389/fendo.2022.899271)
Supplement: Supplementary file 4 [file Table_2.docx]

**Supplementary Table 2:** Pathways activated and suppressed in the EIU rats induced by LPS followed by GTE treatment as analyzed by Metascape in (a) the plasma, and (b) the retina. +: activation; -: suppression

(a)

| **The pathways affected by LPS in the plasma** | **Activation/Suppression** |
| --- | --- |
| Arachidonic acid metabolism | + |
| C21-steroid hormone biosynthesis and metabolism | + |
| Glycerophospholipid metabolism | - |
| Glycine, serine, alanine and threonine metabolism | + |
| Leukotriene metabolism | + |
| Linoleate metabolism | + |
| Lipoate metabolism | + |
| Prostaglandin formation from arachidonate | + |
| Prostaglandin formation from dihomo gama-linoleic acid | + |
| Tyrosine metabolism | - |
|  |  |
| **The pathways relieved by GTE in the plasma** |  |
| Arachidonic acid metabolism | - |
| Bile acid biosynthesis | - |
| De novo fatty acid biosynthesis | - |
| Glycerophospholipid metabolism | + |
| Linoleate metabolism | - |

(b)

| **The pathways affected by LPS in the retina** | **Activation/Suppression** |
| --- | --- |
| Fructose and mannose metabolism | - |
| Glycerophospholipid metabolism | - |
| Glycine, serine, alanine and threonine metabolism | + |
| Lysine metabolism | + |
| Prostaglandin formation from arachidonate | + |
| Pyrimidine metabolism | - |
| Selenoamino acid metabolism | - |
| Tryptophan metabolism | + |
| Urea cycle and metabolism of arginine, proline, glutamate, aspartate and asparagine | + |
| Vitamin B5 - CoA biosynthesis from pantothenate | + |
| Vitamin B6 (pyridoxine) metabolism | - |
|  |  |
| **The pathways relieved by GTE in the retina** |  |
| 3-oxo-10R-octadecatrienoate beta-oxidation | - |
| Glycerophospholipid metabolism | - |
| Glycosphingolipid biosynthesis - ganglioseries | + |
| Methionine and cysteine metabolism | - |
| Prostaglandin formation from arachidonate | - |
| Pyrimidine metabolism | + |
| Vitamin B5 - CoA biosynthesis from pantothenate | - |
