# Supplementary material for: Systemic and Ocular Anti-Inflammatory Mechanisms of Green Tea Extract on Endotoxin-Induced Ocular Inflammation
Source: Front Endocrinol (Lausanne). 2022 Jul 15;13:899271. doi: 10.3389/fendo.2022.899271 (PMC9335207; doi:10.3389/fendo.2022.899271)
Supplement: Supplementary file 5 [file Table_3.docx]

**Supplementary Table 3.** Correlation of plasma metabolites with retinal metabolites. Tables showed (a) correlation coefficients of expression of metabolites in the plasma (p < 0.05, r > 0.4 or r <-0.4) with metabolites expressed in the retina following LPS induction; and (b) correlation coefficients of expression of metabolites expressed in the retina with GTE treatment after LPS induction. ↑: increased metabolite levels in plasma. ↓: decreased metabolite levels in plasma.

(a)

(b)
